# Associations of accelerometer-measured physical activity and sedentary time with chronic kidney disease: The Framingham Heart Study

**DOI:** 10.1371/journal.pone.0234825

**Published:** 2020-06-15

**Authors:** Joowon Lee, Maura E. Walker, Kelley P. Gabriel, Ramachandran S. Vasan, Vanessa Xanthakis

**Affiliations:** 1 Section of Preventive Medicine, Department of Medicine, Boston University School of Medicine, Boston, MA, United States of America; 2 Department of Epidemiology, School of Public Health, The University of Alabama at Birmingham, Birmingham, AL, United States of America; 3 Department of Epidemiology, Boston University School of Public Health, Boston, MA, United States of America; 4 Framingham Heart Study, Framingham, MA, United States of America; 5 Department of Biostatistics, Boston University School of Public Health, Boston, MA, United States of America; International University of Health and Welfare, School of Medicine, JAPAN

## Abstract

**Background:**

Few studies examined the individual and conjoint associations of accelerometer-measured physical activity (PA) and sedentary times with the prevalence of chronic kidney disease (CKD) among older adults.

**Methods:**

We evaluated 1,268 Framingham Offspring Study participants (mean age 69.2 years, 53.8% women) between 2011 and 2014. CKD was defined as an estimated glomerular filtration rate (eGFR) <60 ml/min/1.73^2^ and/or urine albumin-to-creatinine ratio (UACR) ≥25/35 μg/mg (men/women). We used multivariable logistic regression models to relate time spent being sedentary and active with the odds of CKD. We then performed compositional data analysis to estimate the change in the eGFR and UACR when a fixed proportion of time in one activity behavior (among the following: moderate to vigorous physical activity [MVPA], light intensity physical activity [LIPA], and sedentary) is reallocated to another activity behavior.

**Results:**

Overall, 258 participants had prevalent CKD (20.4%; 120 women). Higher total PA ([MVPA+LIPA], adjusted-odds ratio [OR] per 30 minutes/day increase, 0.86; 95% CI, 0.78–0.96) and higher LIPA (OR per 30 minutes/day increase, 0.87; 95% CI, 0.76–0.99) were associated with lower odds of CKD. Additionally, higher sedentary time (OR per 30 minutes/day increase, 1.16; 95% CI, 1.04–1.29) was associated with higher odds of CKD. Reallocating 5% of the time from LIPA to sedentary was associated with the largest predicted difference in eGFR (-1.06 ml/min/1.73m^2^). Reallocating 1% of time spent in MVPA to sedentary status predicted the largest difference in UACR (14.37 μg/mg).

**Conclusion:**

The findings suggest that increasing LIPA and maintaining MVPA at the expense of sedentary time may be associated with a lower risk of CKD in community-based older adults.

## Introduction

The prevalence of chronic kidney disease (CKD) is approximately 13% in the U.S. general population [1] and the number of patients with kidney failure treated by dialysis and transplantation has sharply increased during the past two decades [2]. Despite the efforts focused on prevention, the prevalence of CKD is projected to reach 2 million by 2030 [3]. Additionally, the presence of CKD is prospectively associated with higher rates of cardiovascular disease (CVD) morbidity and mortality [4–6]. Therefore, prevention of early stages of CKD may also have a potential impact on CVD morbidity and mortality, rendering it an issue of public health importance [7].

Overall, evidence consistently suggests that higher physical activity (PA) is associated with lower odds of CKD. Specifically, cross-sectional studies have reported that higher levels of light-intensity PA (LIPA) [8, 9], moderate to vigorous PA (MVPA) [9–13], and total PA (MVPA+LIPA) [8, 14] were associated with lower odds of CKD. Additionally, cross-sectional studies have reported that higher levels of sedentary time are associated with higher odds of CKD in middle-aged [13, 14] and older adults [15]. However, evidence on the association between PA and CKD in older adults has been primarily limited to self-reported PA, which is prone to measurement bias [16]. Importantly, prior studies have been limited to analyses based on standard multivariable regression models, which assume that time spent in one specific activity behavior is independent of time spent in other activity behaviors, when in fact these behaviors are co-dependent when conceptualized as behaviors with respect to the given total (100%) of daily activity time [17]. In other words, reducing the time spent in one behavior (e.g., active time) results in more time in a different behavior (e.g., sedentary time).

The purpose of the current investigation was to: (1) evaluate the individual associations of objectively-measured PA behaviors (Total PA, LIPA, MVPA, and adherence to PA guidelines) and sedentary time with the presence of CKD among older adults using standard multivariable logistic regression analysis; and (2) determine the combined association of time (as a percentage of total daily activity) spent in activity behaviors (MVPA, LIPA, and sedentary) with indicators of kidney function using compositional data analysis in community-based older adults.

## Methods

### Study design and sample

In 1971, the Framingham Offspring Study (FOS) was established with the enrollment of 5,124 women and men who were children of the original cohort participants or the spouses of those children [18]. FOS participants who attended the ninth examination cycle (2011–2014) and agreed to wear an accelerometer were eligible for inclusion in the present investigation. Of the 2,430 participants who attended the ninth examination cycle, 1,162 participants were excluded for the following reasons: refusal to wear an accelerometer (n = 789), invalid PA data (n = 352), unavailable data on serum creatinine (estimated glomerular filtration rate [eGFR]) or spot urine sample (urine-albumin-to-creatinine ratio [UACR]) (n = 7) at the ninth examination cycle, and missing covariates (n = 14), resulting in a final sample size of 1,268 participants. The study was approved by the Boston University Medical Center institutional review board, and all participants provided written informed consent.

### Physical activity measurements

All participants were asked to wear an omnidirectional accelerometer (Actical model no. 198‐0200‐00; Philips Respironics, Murrysville, PA, USA) on the hip for eight days (except when bathing or involved in water activity). This accelerometer records signals within 0.5–3 Hz and accelerations/decelerations within 0.05–2 g. Recorded signals were grouped into ‘counts’ during the 30-second intervals (epoch). Data were analyzed at the Framingham Heart Study using customized software (Kinesoft, version 3.3.63, Saskatchewan, Canada) and a pre‐defined protocol for quality control [19]. Measures from the first day of wear were excluded from the analysis. Accelerometer data were considered valid if the device was worn for ≥10 hours per day for at least 4 days. Non‐wear time was defined as 60 consecutive minutes of zero counts, allowing for 2‐minute interruption periods. In the present investigation, sedentary time was classified as 0–99 counts per epoch, while PA was classified as light (100–742 counts per epoch), moderate (743–2778 counts per epoch), or vigorous (≥2779 counts per epoch) intensity using established cut-points [19, 20]. Each intensity category of PA, including LIPA, moderate PA, and vigorous PA, corresponds to 1.6–3 MET (metabolic equivalent of task), 3–6 MET, and ≥6 MET, respectively, where 1 MET is assumed to be 3.5 ml/kg/min. MVPA was defined as ≥743 counts per epoch. We also analyzed the association of adherence to the 2018 PA guidelines with the odds of CKD. A binary adherence to the 2018 PA guidelines was defined as ≥150 minutes of MVPA/week vs. <150 minutes of MVPA/week. Total PA was the sum of LIPA and MVPA. For participants with <7, but ≥4 days of valid wear time, we averaged the MVPA over the valid days and extrapolated this value across 7 days to estimate MVPA minutes/week.

### Chronic kidney disease

In the present investigation, CKD was defined as an eGFR <60 ml/min/1.73^2^ and/or urine albumin-to-creatinine ratio (UACR) ≥25/35 μg/mg (men/women) at examination nine. This definition is in accordance with the established NKF-KDOQI (National Kidney Foundation’s Kidney Disease Outcomes Quality Initiative) criteria [21]. Serum creatinine was measured using the modified Jaffé method (Colorimetric Assay, Roche Diagnostics). Serum creatinine measures can vary widely across different laboratories. Therefore, we used a two-step serum creatinine calibration process: (1) calibration of National Health and Nutrition Examination Survey III (NHANES III) creatinine values to the Cleveland Clinic Laboratory resulting in average difference in serum creatinine calibration of 0.23 mg/dl (Correction factor), and (2) alignment of mean serum creatinine values from the FOS by sex-specific age groups (20–39, 40–59, 60–69, and ≥ 70 years) with the corresponding corrected NHANES III age- and sex-specific means [22]. The CKD-Epidemiology Collaboration (CKD-EPI) equation was used to estimate the GFR [23]. Spot urine samples were collected at examination cycle nine and stored at −20°C. The urinary albumin concentration was assayed using immunoturbidimetry (Tina Quant Albumin Assay, Roche Diagnostics). Urinary creatinine was measured using the modified Jaffé method (Colorimetric Assay, Roche Diagnostics).

### Covariates

At the ninth examination cycle, covariates were collected from routine medical history, physical examination, and laboratory assessment. In the present investigation, age, sex, accelerometer wear time, current smoking status, body mass index (BMI), total cholesterol: high-density lipoprotein cholesterol ratio (TC/HDL-C), systolic blood pressure (SBP), diabetes (defined as fasting glucose level ≥126 mg/dL or use of antidiabetic medications), use of anti-hypertensive medication, use of lipid-lowering medication, and prevalence of CVD were considered as covariates, and were selected based on their reported previous associations with CKD and PA measurements [8, 14, 24]. CVD includes fatal or nonfatal myocardial infarction, unstable angina (prolonged ischemic episode with documented reversible ST-segment changes), peripheral vascular disease (intermittent claudication), cerebrovascular disease (ischemic or hemorrhagic stroke or transient ischemic attack), or heart failure.

### Statistical methods

We used multivariable-adjusted logistic regression models to estimate odds ratios (OR) and their 95% confidence intervals (CI) for the individual association of time spent in sedentary and PA behaviors (by intensity category; independent variables, separate model for each) with odds of CKD. Analyses were performed using two models: (1) adjusted for age, sex, and accelerometer wear time; and (2) additionally adjusted for current smoking status, BMI, TC/HDL-C, SBP, diabetes, use of anti-hypertensive, use of lipid-lowering medications, and prevalence of CVD at ninth examination cycle. Self-rated health status (four categories ranging from “poor” to “excellent,” derived from the self-administered Short Form [SF]-12 Health Survey question) was used to assess reverse causality. We also examined the interaction of sedentary and PA behaviors with age (dichotomized at the median value) and sex on the prevalence of CKD.

To examine the combined associations of time spent in sedentary and PA behavior with indicators of kidney function, compositional data analysis [17] was conducted using the R package ‘compositions’ [25]. We expressed sedentary time, LIPA, and MVPA as proportions of total activity time (100%) in a day. Data underwent an isometric log-ratio transformation and were represented as isometric log-ratio co-ordinates. The log-ratio transformation allows the use of standard statistical methods on transformed data and subsequent translation of results back into the original units [17]. Geometric means of each composition element were calculated and adjusted to a sum of 100% to determine the average proportion per day engaged in each respective activity behavior. A variation matrix was calculated to determine the variation between composition elements. Lower values in the variation matrix imply greater proportionality (e.g., co-dependence) of two elements. We generated multivariable linear regression models to determine the associations of the total activity composition (all isometric log-ratio coordinates as the independent variable) and individual composition component (individual isometric log-ratio coordinates) with eGFR and UACR (dependent variables; separate model for each) after adjusting for the same covariates as we adjusted for in Model 2, with the exception of accelerometer wear time. Accelerometer wear time was not adjusted in the model because we examined the PA behaviors as a proportion of total PA time (accelerometer wear time) in the compositional data analysis. We evaluated the estimated effect of reallocating time from one activity behavior to another on eGFR and UACR. First, we estimated the mean values of eGFR and UACR using multivariable linear regression models which included the mean proportion of time spent in the respective behaviors (separate models for eGFR and UACR). We then estimated the mean values of eGFR and UACR from a model with time reallocated among activity behaviors (separate model for each time reallocation). Lastly, the estimated difference in eGFR and UACR values for each time reallocation was calculated by subtracting the estimated mean values from each estimated time reallocation value.

A 2-sided value of *P ≤*0.05 was considered statistically significant for all models. All analyses were performed using SAS software version 9.4 (SAS Institute Inc, Cary, NC) and R version 3.5.1 run on RStudio (version 1.0.153, RStudio: Integrated Development for R. RStudio, Inc., Boston, MA).

## Results

Baseline characteristics of the study participants are presented in **Table 1.** Overall, the mean age of participants was 69±7 years and 258 participants had prevalent CKD (20.3%; 120 women) at examination cycle nine. Additionally, 163 participants had prevalent CVD (12.9%; 63 women) at the same examination cycle. Participants who agreed to wear an accelerometer (n = 1,641) were younger and had lower BMI and SBP but higher HDL-C, LDL-C, and diastolic blood pressure (DBP) compared with those who did not wear the accelerometer (n = 789; **S1 Table**). Only two participants reported poor general health (data are not shown). The description of kidney function among participants is presented in **S2 Table**.

**Table 1:** Characteristics of study sample.

Variables	Men (n = 586)	Women (n = 682)
**Clinical characteristics**		
Age (years)	69±8	69±7
BMI (kg/m^2^)	28.5±4.2	27.1±5.2
Total cholesterol (mg/dL)	172±34	198±36
HDL-C (mg/dL)	55±15	69±18
LDL-C (mg/dL)	94±29	106±32
Triglycerides (mg/dL)	109±54	110±52
Use of lipid lowering medications (n, %)	315 (53.8)	284 (41.6)
SBP (mm Hg)	127±15	125±16
DBP (mm Hg)	74±9	71±9
Hypertension (n, %)	362 (61.8)	368 (54.0)
Use of antihypertensive medications (n, %)	325 (55.5)	311 (45.6)
Fasting glucose (mg/dL)	106±23	98±15
Diabetes (n, %)	88 (15.0)	54 (7.9)
Current Smoking (n, %)	20 (3.4)	47 (6.9)
eGFR <60 ml/min/1.73m^2^ (n, %)	98 (16.7)	96 (14.1)
UACR ≥25/35 μg/mg (men/women) (n, %)	59 (10.1)	34 (5.0)
Presence of CVD (n, %)	100 (17.1)	63 (9.2)
Presence of CKD (n, %)	138 (23.6)	120 (17.6)
**Physical activity variables**		
Accelerometer Wear Time (min/day)	791.6±77.9	788.0±73.3
Total PA (min/day)	131.6±59.5	113.0±50.9
LIPA (median [25^th^, 75^th^ percentile], min/day)	107.3 (78.8, 136.5)	95.2 (70.0, 120.4)
MVPA (median [25^th^, 75^th^ percentile], min/day)	14.1 (6.0, 28.5)	8.9 (2.5, 20.8)
Sedentary Time (median [25^th^, 75^th^ percentile], min/day)	658.4 (612.5, 705.4)	668.8 (628.3, 718.1)
Adherence to PA Guidelines (≥150 min/week, %)	214 (36.5)	164 (24.1)

**Abbreviations:** BMI, body mass index; HDL-C, high-density lipoprotein cholesterol; LDL-C, low-density lipoprotein cholesterol; SBP, systolic blood pressure; DBP, diastolic blood pressure; eGFR, estimated glomerular filtration rate; UACR, urine albumin to creatinine ratio; CVD, cardiovascular disease; CKD, chronic kidney disease; PA, physical activity, LIPA, light intensity physical activity; MVPA, moderate to vigorous physical activity.

CVD includes fatal or nonfatal myocardial infarction, unstable angina (prolonged ischemic episode with documented reversible ST-segment changes), peripheral vascular disease (intermittent claudication), cerebrovascular disease (ischemic or hemorrhagic stroke or transient ischemic attack), or heart failure; Values are mean±SD unless otherwise indicated.

### Individual associations of physical activity and sedentary time with the presence of CKD

The individual associations of PA and sedentary time with odds of CKD are shown in **Table 2**. All PA variables were inversely associated with odds of CKD in unadjusted models (Model 1) In the partially-adjusted models (Model 2: adjustment for age, sex, and accelerometer wear time), higher total PA (per 30 minutes/day increase), MVPA (per 10 minutes/day increase), and LIPA (per 30 minutes/day increase) were significantly associated with lower odds of CKD, whereas higher sedentary time (per 30 minutes/day increase) was significantly associated with higher odds of CKD. Associations were slightly attenuated after further adjustment for smoking, BMI, TC/HDL-C, SBP, use of antihypertensive medication, diabetes, use of lipid-lowering medication, and prevalence of CVD. The inverse association between MVPA and odds of CKD was attenuated and no longer statistically significant in the fully-adjusted model (Model 3). We did not observe significant effect modifications in the associations of sedentary and PA behaviors with CKD by age or sex (data not shown).

**Table 2:** Individual associations between objectively measured physical activity and prevalence of CKD.

			Model 1		Model 2		Model 3	
			OR (95% CI)	P-value	OR (95% CI)	P-value	OR (95% CI)	P-value
Total PA (per 30 min/day increase)			0.71 (0.64–0.77)	< .001	0.82 (0.74–0.92)	< .001	0.86 (0.78–0.96)	.007
MVPA (per 10 min/day increase)			0.76 (0.69–0.84)	< .001	0.87 (0.79–0.96)	.005	0.92 (0.84–1.01)	.07
PA Guideline (MVPA ≥150 min/week or not)			0.50 (0.36–0.70)	< .001	0.71 (0.50–1.03)	.07	0.89 (0.60–1.31)	.55
LIPA (per 30 min/day increase)			0.68 (0.61–0.76)	< .001	0.83 (0.73–0.94)	.004	0.87 (0.76–0.99)	.03
Sedentary time (per 30 min/day increase)			1.04 (0.99–1.10)	.14	1.22 (1.09–1.35)	< .001	1.16 (1.04–1.29)	.007

**Abbreviations:** CKD, chronic kidney disease; OR, odds ratio; CI, confidence interval; PA, physical activity; MVPA, moderate to vigorous physical activity; LIPA, light intensity physical activity.

Model 1 is unadjusted; Model 2 adjusted for age, sex, and accelerometer wear time; Model 3 adjusted for age, sex, accelerometer wear time, smoking, BMI, SBP, use of antihypertensive medication, diabetes, TC/HDL-C, use of lipid-lowering medication, and the prevalence of CVD at exam 9; All PA variables and sedentary time were considered as exposure variables (separate model for each); CVD includes fatal or nonfatal myocardial infarction, unstable angina (prolonged ischemic episode with documented reversible ST-segment changes), peripheral vascular disease (intermittent claudication), cerebrovascular disease (ischemic or hemorrhagic stroke or transient ischemic attack), or heart failure.

### Compositional data analysis

On average, participants spent 86.6% of their total daily activity time being sedentary, 12.2% of their activity time in LIPA, and 1.2% of time in MVPA. We observed a high co-dependency between the proportions of time spent being sedentary and in LIPA (**Table 3**). The proportion of time spent in MVPA exhibited the lowest co-dependency with other activity behaviors. Model parameters from multivariable linear regression indicated that isometric log-ratio co-ordinates (total composition; single variable), age, TC/HDL-C, use of lipid-lowering medication, use of anti-hypertensive medication, and prevalence of CVD were statistically significant predictors of eGFR (**S3 Table**). Additionally, isometric log-ratio co-ordinates (total composition, single variable), TC/HDL-C, diabetes, and prevalence of CVD were statistically significant predictors for UACR (**S4 Table**).

**Table 3:** Compositional variation matrix of time spent in individual activity behaviors.

	LIPA	MVPA
Sedentary	0.24	2.31
LIPA	-	1.69
MVPA	-	-

**Abbreviations**: LIPA, light intensity physical activity; MVPA, moderate to vigorous physical activity.

Values closer to zero indicate that the proportion of time in the two physical activity behaviors included in the ratio was highly co-dependent.

We examined the hypothetical reallocation of 1% (approximately 8 minutes/day [geometric mean]) and 5% (approximately 39 minutes/day [geometric mean]) of total activity time between LIPA and sedentary behaviors on eGFR and UACR. On average, participants spent approximately 1% of total activity time in MVPA so we did not test the hypothetical reallocation of 5% of the time from MVPA to other activity behaviors. Further, the reallocation of 5% from other activity behaviors to MVPA may not be an achievable behavioral target in an older adult population. All compositional activity behaviors (expressed as individual isometric log-ratio co-ordinates) were significantly associated (*P* <0.05) with eGFR. Overall, we observed the largest predicted difference in eGFR following the hypothetical reallocation of 5% of time from LIPA to sedentary (**Table 4**). This resulted in a predicted difference of -1.06 ml/min/1.73m^2^ in eGFR, indicating an inverse association between time spent sedentary and eGFR. The opposite time reallocation, 5% from sedentary to LIPA, predicted a favorable 0.73 ml/min/1.73m^2^ increase in eGFR. The reallocation of 1% of the time from sedentary to MVPA was associated with a 0.06 ml/min/1.73m^2^ increase in eGFR. This association was asymmetrical with a 1% reallocation of time from MVPA to sedentary time resulting in a 0.13 ml/min/1.73m^2^ decrease in eGFR. The association between the proportion of time spent sedentary and in LIPA (expressed as individual isometric log-ratio co-ordinates) had marginal statistically significant (*P* <0.05) associations with UACR. The proportion of time spent in MVPA (expressed as an individual isometric log-ratio co-ordinate) had a highly significant association (*P* <0.01) with UACR. The largest predicted differences for UACR was observed with the reallocation of 1% of total activity time from MVPA to sedentary (**Table 4**). This was associated with a predicted unfavorable increase of 14.37 μg/mg in UACR. These associations were asymmetrical with the reallocation of time from sedentary state leading to disproportionately smaller favorable decreases (4.82 μg/mg) in UACR. The reallocation of 5% of the time from sedentary to LIPA was associated with a 10.27 μg/mg decrease in UACR. The opposite reallocation (5% of the time from LIPA to sedentary) predicted an increase in UACR of 14.27 μg/mg.

**Table 4:** Predicted differences in eGFR (ml/min/1.73m^2^) and UACR (μg/mg) following reallocation of physical activity time.

**eGFR**	Sedentary time	LIPA	MVPA
Sedentary time (1%)	-	0.16	0.06
LIPA (1%)	-0.18	-	-0.12
MVPA (1%)	-0.13	0.03	-
Sedentary time (5%)	-	0.73	0.21
LIPA (5%)	-1.06	-	-0.86
**UACR**	Sedentary time	LIPA	MVPA
Sedentary time (1%)	-	-2.23	-4.82
LIPA (1%)	2.39	-	-2.43
MVPA (1%)	14.37	12.14	-
Sedentary time (5%)	-	-10.27	-13.85
LIPA (5%)	14.27	-	0.53

**Abbreviations:** eGFR, estimated glomerular filtration rate; UACR, urine albumin to creatinine ratio; LIPA, light intensity physical activity; MVPA, moderate to vigorous physical activity.

Reallocation of proportions of time from the physical activity behaviors in rows to physical activity behaviors in columns; For example, 0.16 ml/min/1.73m^2^ increase from the mean eGFR if 1% of sedentary time is replaced by 1% of LIPA; All compositional activity behaviors (Sedentary time, LIPA, and MVPA expressed as individual isometric log-ratio co-ordinates) were significantly associated (*P* <0.05) with eGFR and UACR.

## Discussion

### Principal findings

In the current investigation, we examined the individual and conjoint associations of objectively measured PA and sedentary time with the prevalence of CKD using standard regression modeling. Additionally, we estimated the change in eGFR and UACR when a fixed proportion of time in one activity behavior was reallocated to another activity behavior using compositional data analysis. In the multivariable logistic regression analysis, higher total PA and LIPA were associated with lower odds of CKD, while higher sedentary time was directly associated with higher odds of CKD. In addition to these findings, our compositional data analysis indicated that increasing LIPA and maintaining MVPA at the expense of sedentary time had the largest predicted impact on clinical indicators of kidney function (eGFR and UACR). Collectively, the findings from the present investigation highlight the importance of maintaining a physically active lifestyle to maintain kidney function in community-based older adults.

### Comparison with the literature

Consistent with the present investigation, prior studies have documented that higher total PA [8, 13, 14, 24] and LIPA [9, 24] were associated with lower odds of CKD in middle-aged [13, 14, 24] and older adults [9, 26]. Additionally, cross-sectional studies have reported that higher sedentary time was associated with higher odds of CKD [9, 13–15]. Most prior studies assessed PA or sedentary time using a self-reported questionnaire [13, 15, 24, 26], which may introduce measurement bias and influence the precision of the observed relation, and primarily focused on middle-aged adults or older men. Therefore, the findings from the present investigation contribute to the existing literature by demonstrating the significant associations of accelerometer-measured total PA, LIPA, and sedentary time with the odds of CKD among older men and women in the community.

Results from our compositional analysis indicated an inverse association of LIPA with kidney function (eGFR and UACR). Our results suggest that reallocating time to or from LIPA had the greatest impact on change in eGFR, and that reallocating between LIPA and sedentary additionally had a beneficial impact on change in UACR, which provides additional clinically relevant information. These findings suggest that increasing LIPA or maintaining high levels of LIPA, at the expense of sedentary time, may be important for improving or maintaining renal function among older adults. In our investigation, older adults spent approximately 1% of daily activity time engaged in MVPA compared to more than 12% of daily activity time engaged in LIPA, which is comparable to a nationally representative sample of older adults [27]. Additionally, data from several longitudinal cohort studies have documented age-related declines in MVPA, particularly during the mid to late-life transition [28]. Hence, health promotion messaging focused on promoting LIPA and reducing sedentary time may be the most clinically relevant strategy for improving kidney function or CKD prevention in the elderly.

Conflicting results exist with regard to the relations between MVPA and odds of CKD [8, 10–14]. In the present investigation, the association between accelerometer-measured MVPA and odds of CKD was attenuated and no longer statistically significant after further adjustment for CVD risk factors in a standard regression modeling approach. Using compositional data analysis, we observed that time spent in MVPA was associated with favorable changes in kidney function. In particular, the time spent in MVPA was the most important activity behavior in modifying UACR. This analysis additionally revealed that maintaining MVPA (i.e., preventing reallocation of time *from* MVPA) may be more important for kidney function (eGFR and UACR) than engaging in greater MVPA (i.e., reallocation of time to MVPA). Thus, increasing MVPA without considering the time spent in other activity behaviors may not be the major determinant of kidney function in older adults. Hence, discrepancies across studies examining MVPA and CKD may in part be due to the use of standard regression models that do not account for the co-dependence between activity behaviors within a given waking period or differing definitions of CKD (eGFR alone vs. both eGFR and UACR). Although the mechanistic link between PA and CKD is not fully elucidated, prior evidence suggests that improved endothelial function in the kidney vasculature [29], podocyte-specific insulin resistance [30], adiposity distribution [31], and obesity-associated inflammation [32] may serve as an underlying biological mechanism.

### Strengths and limitations

The present investigation has several strengths. Compositional data analysis using the isometric log-ratio transformation methodology allows for the appropriate inference of one activity behavior with respect to all other kinds of activities. Additionally, the use of a large community-based cohort free of CKD at baseline could minimize selection bias. The use of accelerometer-measured PA and sedentary behaviors that may lessen measurement bias is another strength of this investigation. Lastly, a comprehensive and detailed assessment of CVD risk factors in the FOS may reduce the residual confounding in the current investigation.

However, there are also limitations that must be considered when interpreting the results of the current investigation. We used a cross-sectional study design that does not permit causal inferences or conclusions regarding the directionality of the relations between PA behaviors and CKD. However, there were only seven participants with advanced CKD (eGFR≤ 30) and two participants with poor general health assessed by Short-Form 12-Item Health Survey (SF-12) suggesting our findings may not reflect the reverse causality [33]. Our definition of the presence of CKD was based on single occasion measurements of serum and spot urine creatinine at serial quadrennial FOS examinations. Additionally, there were no available data on cystatin C, which is less likely to be influenced by age-related sarcopenia in older adults. Careful consideration is warranted as to whether the PA cut-points used in the present investigation are appropriate for older adults. Evanson et al. have advocated for the use of individualized PA cut-points given that the fitness levels among older adults vary due to health conditions such as frailty [34]. Thus, there may be misclassification of PA behaviors in our sample because of the cut-points used. Selection bias may also be present since the participants who refuse to wear an accelerometer had poorer cardio-metabolic profiles that may be associated with the prevalence of CKD. Lastly, the generalizability of the current investigation is limited by the characteristics of the FOS that consists of white individuals of European ancestry; additional studies of multi-ethnic samples are needed to confirm our observations.

## Conclusion

In the present cross-sectional investigation, we observed that higher total PA and LIPA were associated with lower odds of CKD. Additionally, higher sedentary time was associated with higher odds of CKD in community-based older adults. Our compositional data analysis additionally revealed that increasing LIPA and maintaining MVPA, at the expense of reducing sedentary time, may potentially improve kidney function. Our findings are consistent with public health efforts that target reducing sitting time and increasing LIPA and maintaining MVPA to increase energy expenditure, measures that in turn may maintain kidney function in older people.

## Supporting information

S1 Table Characteristics of participants included and excluded from the analysis. (DOCX)

S2 Table Description of kidney function among participants. (DOCX)

S3 Table A linear model of isometric log-coordinates and eGFR: Analysis of variance. (DOCX)

S4 Table A linear model of isometric log-coordinates and UACR: Analysis of variance. (DOCX)
