# Supplementary material for: Associations of accelerometer-measured physical activity and sedentary time with chronic kidney disease: The Framingham Heart Study
Source: PLoS One. 2020 Jun 15;15(6):e0234825. doi: 10.1371/journal.pone.0234825 (PMC7295223; doi:10.1371/journal.pone.0234825)
Supplement: S1 Table — (DOCX) [file pone.0234825.s001.docx]

**Supplementary Table 1. Characteristics of participants included and excluded from the analysis.**

|  | Included (n=1,641) | Exclude (n=789) |
| --- | --- | --- |
| Age (years) | 70±8 | 74±10 |
| BMI (kg/m^2^) | 28.2±5.1 | 29.1±6.0 |
| Total cholesterol (mg/dL) | 184±38 | 181±38 |
| HDL-C (mg/dL) | 62±19 | 61±20 |
| LDL-C (mg/dL) | 100±31 | 96±30 |
| Triglycerides (mg/dL) | 111±53 | 122±65 |
| Use of lipid lowering medications (n, %) | 808 (49.2) | 422 (53.8) |
| SBP (mm Hg) | 126±16 | 129±17 |
| DBP (mm Hg) | 72±9 | 70±10 |
| Hypertension (n, %) | 872 (53.2) | 517 (65.9) |
| Use of antihypertensive medications (n, %) | 995 (60.7) | 567 (72.3) |
| Fasting glucose (mg/dL) | 103±20 | 106±22 |
| Diabetes (n, %) | 214 (13.2) | 136 (20.6) |
| Current Smoking (n, %) | 86 (5.3) | 55 (7.0) |
| Presence of CVD (n, %) | 240 (14.6) | 213 (27.0) |
| Presence of CKD (n, %) | 392 (24.0) | 198 (31.6) |

**Abbreviations:** BMI, body mass index; HDL-C, high-density lipoprotein cholesterol; LDL-C, low-density lipoprotein cholesterol; SBP, systolic blood pressure; DBP, diastolic blood pressure; CVD, cardiovascular disease; CKD, chronic kidney disease.

**Note:** CVD includes fatal or nonfatal myocardial infarction, unstable angina (prolonged ischemic episode with documented reversible ST-segment changes), peripheral vascular disease (intermittent claudication), cerebrovascular disease (an ischemic or hemorrhagic stroke or transient ischemic attack), or heart failure; Values are mean+SD unless otherwise indicated.
