# Supplementary material for: Associations of accelerometer-measured physical activity and sedentary time with chronic kidney disease: The Framingham Heart Study
Source: PLoS One. 2020 Jun 15;15(6):e0234825. doi: 10.1371/journal.pone.0234825 (PMC7295223; doi:10.1371/journal.pone.0234825)
Supplement: S2 Table — (DOCX) [file pone.0234825.s002.docx]

**Supplementary Table 2.** **Description of kidney function among participants.**

|  | | Total (Mean±SD) | Men (Mean±SD) | Women (Mean±SD) |
| --- | --- | --- | --- | --- |
| eGFR (ml/kg/1.73m^2^) | | 75.8±15.3 | 75.9±15.5 | 75.7±15.1 |
| CKD-G stage | eGFR  (ml/min/1.73m^2^) | Total, n (%) | Men, n (%) | Women, n (%) |
| Stage 1 | ≥90 | 231 (18.2) | 116 (19.8) | 115 (16.9) |
| Stage 2 | 60-89 | 842 (66.5) | 372 (63.5) | 470 (69.0) |
| Stage 3a | 45-59 | 151 (11.9) | 76 (13.0) | 75 (11.0) |
| Stage 3b | 30-44 | 36 (2.8) | 19 (3.2) | 17 (2.5) |
| Stage 4 | 15-29 | 6 (0.5) | 3 (0.5) | 3 (0.4) |
| Stage 5 | <15 | 1 (0.1) | 0 (0.0) | 1 (0.2) |
|  | | Total (Mean±SD) | Men (Mean±SD) | Women (Mean±SD) |
| UACR (mg/g) | | 23.1±223.8 | 19.1±121.4 | 26.6±283.7 |
| CKD-A stage | UACR  (mg/g) | Total, n (%) | Men, n (%) | Women, n (%) |
| 1 | <30 | 1181 (93.2) | 541 (92.3) | 640 (94.0) |
| 2 | 30-300 | 76 (6.0) | 41 (7.0) | 35 (5.1) |
| 3 | >300 | 10 (0.8) | 4 (0.7) | 6 (0.9) |

**Abbreviations:** SD, standard deviation; eGFR, estimated glomerular filtration rate; CKD, chronic kidney disease; UACR, urine albumin to creatinine ratio.
