# Supplementary material for: Associations of accelerometer-measured physical activity and sedentary time with chronic kidney disease: The Framingham Heart Study
Source: PLoS One. 2020 Jun 15;15(6):e0234825. doi: 10.1371/journal.pone.0234825 (PMC7295223; doi:10.1371/journal.pone.0234825)
Supplement: S3 Table — (DOCX) [file pone.0234825.s003.docx]

**Supplementary Table 3. A linear model of isometric log-coordinates and eGFR: analysis of variance**

|  | Sum Sq. | Df | F value | p-value |
| --- | --- | --- | --- | --- |
| Isometric log-ratio co-ordinates | 22017 | 2 | 64.87 | <.001 |
| Age | 53647 | 1 | 316.14 | <.001 |
| Sex | 14 | 1 | 0.08 | .78 |
| BMI | 15 | 1 | 0.09 | .76 |
| Smoking | 364 | 1 | 2.15 | .14 |
| SBP | 203 | 1 | 1.20 | .27 |
| Use of lipid-lowering medication | 852 | 1 | 5.02 | .03 |
| Use of antihypertensive medication | 3337 | 1 | 19.67 | <.001 |
| Diabetes | 24 | 1 | 0.14 | .71 |
| Prevalence of CVD | 2349 | 1 | 13.84 | .002 |
| TC:HDL | 745 | 1 | 4.39 | .04 |

**Abbreviations**: eGFR, estimated glomerular filtration rate; Sum Sq.; sum of squares; DF, degree of freedom; BMI, body mass index; SBP, systolic blood pressure, CVD, cardiovascular disease; TC, total cholesterol; HDL-C, high-density lipoprotein cholesterol.

**Note**: The ANOVA output demonstrates how the entire composition contributes to the model; CVD includes fatal or nonfatal myocardial infarction, unstable angina (prolonged ischemic episode with documented reversible ST-segment changes), peripheral vascular disease (intermittent claudication), cerebrovascular disease (ischemic or hemorrhagic stroke or transient ischemic attack), or heart failure; All variables were included in the same model.
