# Supplementary material for: Associations of accelerometer-measured physical activity and sedentary time with chronic kidney disease: The Framingham Heart Study
Source: PLoS One. 2020 Jun 15;15(6):e0234825. doi: 10.1371/journal.pone.0234825 (PMC7295223; doi:10.1371/journal.pone.0234825)
Supplement: S4 Table — (DOCX) [file pone.0234825.s004.docx]

**Supplementary Table 4. A linear model of isometric log-coordinates and UACR: analysis of variance**

|  | Sum Sq. | Df | F value | p-value |
| --- | --- | --- | --- | --- |
| Isometric log-ratio co-ordinates | 745882 | 2 | 7.58 | <.001 |
| Age | 13135 | 1 | 0.27 | .61 |
| Sex | 1890 | 1 | 0.04 | .84 |
| BMI | 151255 | 1 | 2.07 | .08 |
| Smoking | 11318 | 1 | 0.23 | .63 |
| SBP | 7374 | 1 | 0.15 | .70 |
| Use of lipid-lowering medication | 18264 | 1 | 0.37 | .54 |
| Use of antihypertensive medication | 31701 | 1 | 0.64 | .42 |
| Diabetes | 235564 | 1 | 4.79 | .03 |
| Prevalence of CVD | 25991 | 1 | 5.28 | .02 |
| TC:HDL | 216991 | 1 | 4.41 | .04 |

**Abbreviations**: UACR, urine albumin to creatinine ratio; Sum Sq.; sum of squares; DF, degree of freedom; BMI, body mass index; SBP, systolic blood pressure, CVD, cardiovascular disease; TC, total cholesterol; HDL-C, high-density lipoprotein cholesterol.

**Note**: The ANOVA output demonstrates how the entire composition contributes to the model; CVD includes fatal or nonfatal myocardial infarction, unstable angina (prolonged ischemic episode with documented reversible ST-segment changes), peripheral vascular disease (intermittent claudication), cerebrovascular disease (ischemic or hemorrhagic stroke or transient ischemic attack), or heart failure; All variables were included in the same model.
